# DNA barcoding reveals twelve lineages with properties of phylogenetic and biological species within *Melitaea didyma* sensu lato (Lepidoptera, Nymphalidae)

**DOI:** 10.3897/zookeys.538.6605

**Published:** 2015-11-19

**Authors:** Elena A. Pazhenkova, Evgeny V. Zakharov, Vladimir A. Lukhtanov

**Affiliations:** 1 Department of Karyosystematics, Zoological Institute of the Russian Academy of Sciences, Universitetskaya emb. 1, 199034 St. Petersburg, Russia; 2 Department of Entomology, Faculty of Biology, St. Petersburg State University, Universitetskaya emb. 7/9, 199034 St. Petersburg, Russia; 3 Biodiversity Institute of Ontario, University of Guelph, Guelph, ON, Canada N1G 2W1; 4 McGuire Center for Lepidoptera and Biodiversity, Florida Museum of Natural History, McGuire Hall, 3215 Hull Road, PO Box 112710, University of Florida, Gainesville FL 32611-2710, USA

**Keywords:** Biodiversity, butterflies, *COI*, cryptic species, mitochondrial DNA, Nymphalidae, phylogeography, taxonomy

## Abstract

The complex of butterfly taxa close to *Melitaea didyma* includes the traditionally recognized species *Melitaea didyma*, *Melitaea didymoides* and *Melitaea sutschana*, the taxa that were recognized as species only relatively recently (*Melitaea latonigena*, *Melitaea interrupta*, *Melitaea chitralensis* and *Melitaea mixta*) as well as numerous described subspecies and forms with unclear taxonomic status. Here analysis of mitochondrial DNA barcodes is used to demonstrate that this complex is monophyletic group consisting of at least 12 major haplogroups strongly differentiated with respect to the gene *COI*. Six of these haplogroups are shown to correspond to six of the above-mentioned species (*Melitaea didymoides*, *Melitaea sutschana*, *Melitaea latonigena*, *Melitaea interrupta*, *Melitaea chitralensis* and *Melitaea mixta*). It is hypothesized that each of the remaining six haplogroups also represents a distinct species (*Melitaea mauretanica*, *Melitaea occidentalis*, *Melitaea didyma*, *Melitaea neera*, *Melitaea liliputana* and *Melitaea turkestanica*), since merging these haplogroups would result in a polyphyletic assemblage and the genetic distances between them are comparable with those found between the other six previously recognized species.

## Introduction

The complex of butterfly taxa close to *Melitaea didyma* (Esper, 1779) is widely distributed in the Palaearctic region. This complex includes the traditionally recognized species *Melitaea didyma*, *Melitaea didymoides* Eversmann, 1847 and *Melitaea sutschana* Staudinger, 1892, the taxa that were recognized as species only recently (*Melitaea latonigena* Eversmann, 1847, *Melitaea interrupta* Colenati, 1846, *Melitaea chitralensis* Moore, 1901 and *Melitaea mixta* Evans, 1912) as well as numerous described subspecies and forms with unclear taxonomic status (Higgins 1941, 1955, Hesselbarth et al. 1995, Kolesnichenko 1999, Kolesnichenko et al. 2011). All these taxa are similar in male and female wing pattern and genitalia structure (Higgins 1941). In our opinion, this complex does not include the species *Melitaea deserticola* Oberthür, 1909, *Melitaea ala* Staudinger, 1881, *Melitaea enarea* Frühstorfer, 1917 and *Melitaea persea* Kollar, 1849 which are similar to *Melitaea didyma* in wing color and pattern but were shown to be distinctly different with respect to genitalia structure (Higgins 1941). The first significant review of this complex was published by Higgins (1941, 1955) in frame of a complete revision of the genus *Melitaea*. Recently the genus *Melitaea* was revised by Oorschot and Coutsis (2014). The taxa within the *Melitaea didyma* complex have a strong morphological variation between individuals of different generations and indistinct clinal variability in wing size and color from north to south (Lvovsky and Morgun 2007). Available cytogenetic (Lukhtanov and Kuznetsova 1989), morphological (Lvovsky and Morgun 2007, Kolesnichenko et al. 2011, Oorschot and Coutsis 2014) and molecular (Wahlberg and Zimmermann 2000, Lukhtanov et al. 2009, Dincă et al. 2015) data show that the *Melitaea didyma* species complex requires a more detailed taxonomic revision.

Here analysis of mitochondrial DNA barcodes is used to demonstrate that this complex is a natural (monophyletic) group consisting of at least 12 major haplogroups strongly differentiated with respect to the gene *COI*. Then the taxonomy of the *Melitaea didyma* species complex is discussed.

## Material and methods

Standard *COI* barcodes (658-bp 5’ segment of mitochondrial *cytochrome oxidase subunit I*) were studied. *COI* sequences were obtained from 85 specimens collected in Afghanistan, Armenia, Austria, Bulgaria, China, Israel, Kazakhstan, Kyrgyzstan, Mongolia, Morocco, Russia, Syria, Tajikistan, Turkey and Uzbekistan. Collection data of these samples are presented in the Suppl. material 1.

Legs from 24 specimens (KT792884–KT792908, see the Suppl. material 2) were processed at the Department of Karyosystematics of the Zoological Institute of the Russian Academy of Sciences. The set of voucher specimens of these butterflies is kept in the Zoological Institute of the Russian Academy of Science (St. Petersburg). DNA was extracted from a single leg removed from each voucher specimen. For DNA extraction we used the GeneJet Genomic DNA Purification Kit (Fermentas) in accordance with the manufacturer’s instructions. Extracted DNA samples were stored at -20 °C.

For DNA amplification we used primers LepF 5’- ATTCAACCAATCATAAAGATATTGG-3’ and LepR (5’-TAAACTTCTGGATGTCCAAAAAATCA-3’ (deWaard et al. 2008). The polymerase chain reaction (PCR) was carried out in 25-mL reactions using a DNA Engine thermal cycler (Eppendorf Mastercycler personal), and typically contained 0.5 mM of each primer, 0.8 mM dNTPs, Fermentas PCR buffer with additional MgCl2 to a final concentration of 2 mM and 1.25 units Fermentas Taq DNA polymerase. All reactions were initially denatured at 94 °C for 2 min, and then subjected to 30 cycles of 60 s at 94 °C denaturation, 60 s at 47 °C and 90 s at 72 °C extension. After amplification, double-stranded DNA was purified using GeneJet PCR Purification Kit (Fermentas). Sequencing of double-stranded product was carried out at the Research Resource Center for Molecular and Cell Technologies.

Legs from 61 specimens of *Melitaea* (HM404715–HM404718, KT874693–KT874751, see the Suppl. material 2) were processed at the Canadian Centre for DNA Barcoding (CCDB, Biodiversity Institute of Ontario, University of Guelph) using standard high-throughput protocol described in deWaard et al. (2008). The set of voucher specimens of these butterflies is kept at the McGuire Center for Lepidoptera and Biodiversity (University of Florida), at the Zoological Institute of the Russian Academy of Science (St. Petersburg) and in Museum for Insects, Pyatigorsk, Russia (Suppl. material 1).

The analysis involved 148 *COI* sequences (including outgroup). Among them there were 63 published sequences (Wahlberg and Zimmermann 2000, Vila and Bjorklund 2004, Leneveu et al. 2009, Lukhtanov et al. 2009, Dincă et al. 2011, 2015, Hausmann et al. 2011, Ashfaq et al. 2013) collected from GenBank (Suppl. material 2). Sequences were aligned using BioEdit software (Hall 1999) and edited manually. Phylogenetic hypotheses were inferred using Bayesian inference (BI), maximum-likelihood (ML) and maximum-parsimony (MP) analyses as described previously (Vershinina and Lukhtanov 2010, Talavera et al. 2013a). Briefly, Bayesian analyses were performed using the program MrBayes 3.1.2 (Huelsenbeck and Ronquist 2001) with default settings as suggested by Mesquite (Maddison and Maddison 2015): burn-in=0.25, nst=6 (GTR + I + G). Two runs of 10,000,000 generations with four chains (one cold and three heated) were performed. Chains were sampled every 10,000 generations. The average value of the Potential Scale Reduction Factor (PSRF) was 1.002 and average standard deviation of split frequencies was 0.01492, to the end of the analysis indicating that convergence was achieved, and a good sample from the posterior probability distribution was obtained.

The ML trees were inferred by using MEGA6 (Tamura et al. 2013) with the nucleotide substitution model T92 (Tamura 1992) as suggested by jModelTest (Posada 2008).

MP analysis was performed using a heuristic search as implemented in MEGA6 (Tamura et al. 2013). A heuristic search was carried out using the close-neighbour-interchange algorithm with search level 3 (Nei and Kumar 2000) in which the initial trees were obtained with the random addition of sequences (100 replicates). We used non-parametric bootstrap values (Felsenstein 1985) to estimate branch support on the reconstructed ML and MP tree. Branch support was assessed using 1000 bootstrap replicates.

## Results and discussion

**Figure 1.**
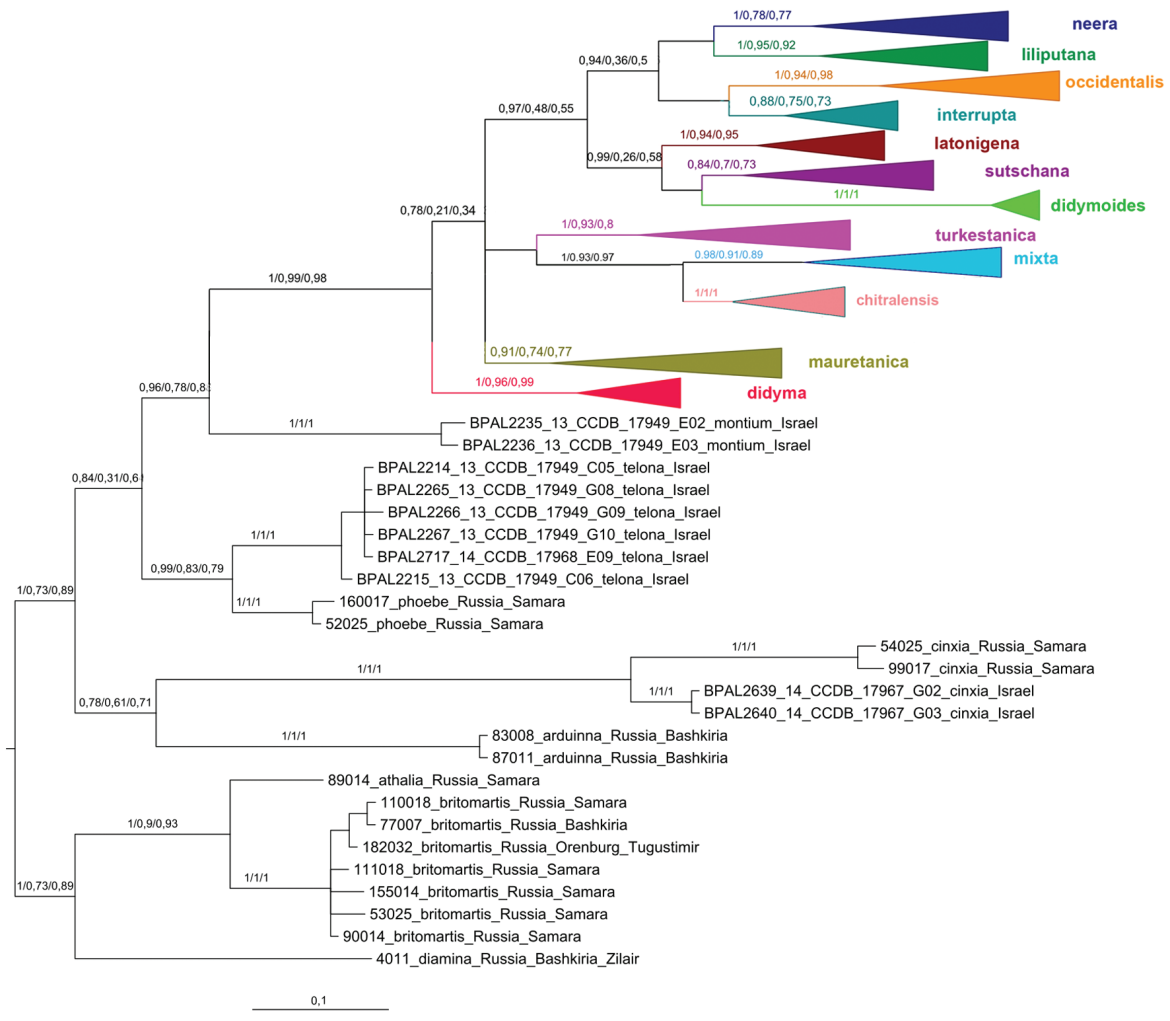
The Bayesian tree of *Melitaea* based on analysis of *the cytochrome oxidase subunit I*
(COI) gene. Numbers at nodes indicate Bayesian posterior probability/ML bootstrap/MP bootstrap values. Scale bar = 0.1 substitutions per position.

**Figure 2.**
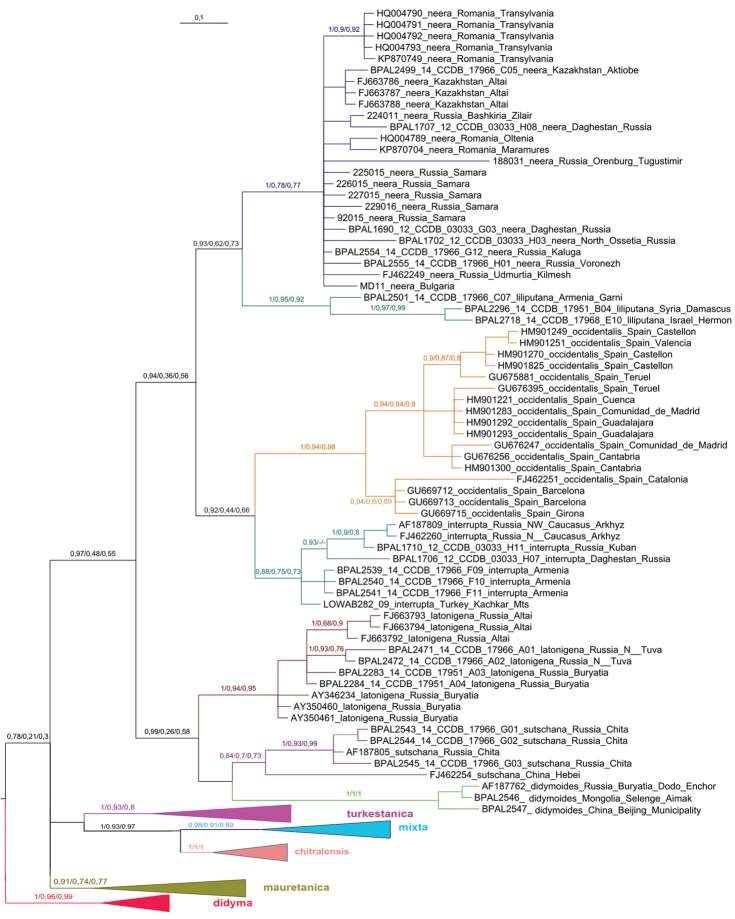
Fragment of the Bayesian tree of *Melitaea didyma* complex (haplogroups *neera*, *liliputana*, *occidentalis*, *interrupta*, *latonigena*, *sutschana* and *didymoides*) based on analysis of the *cytochrome oxidase subunit I*
(COI) gene. Numbers at nodes indicate Bayesian posterior probability/ML bootstrap/MP bootstrap values, with nonmatching clades using different analyses indicated by ‘-’. Scale bar = 0.1 substitutions per position.

**Figure 3.**
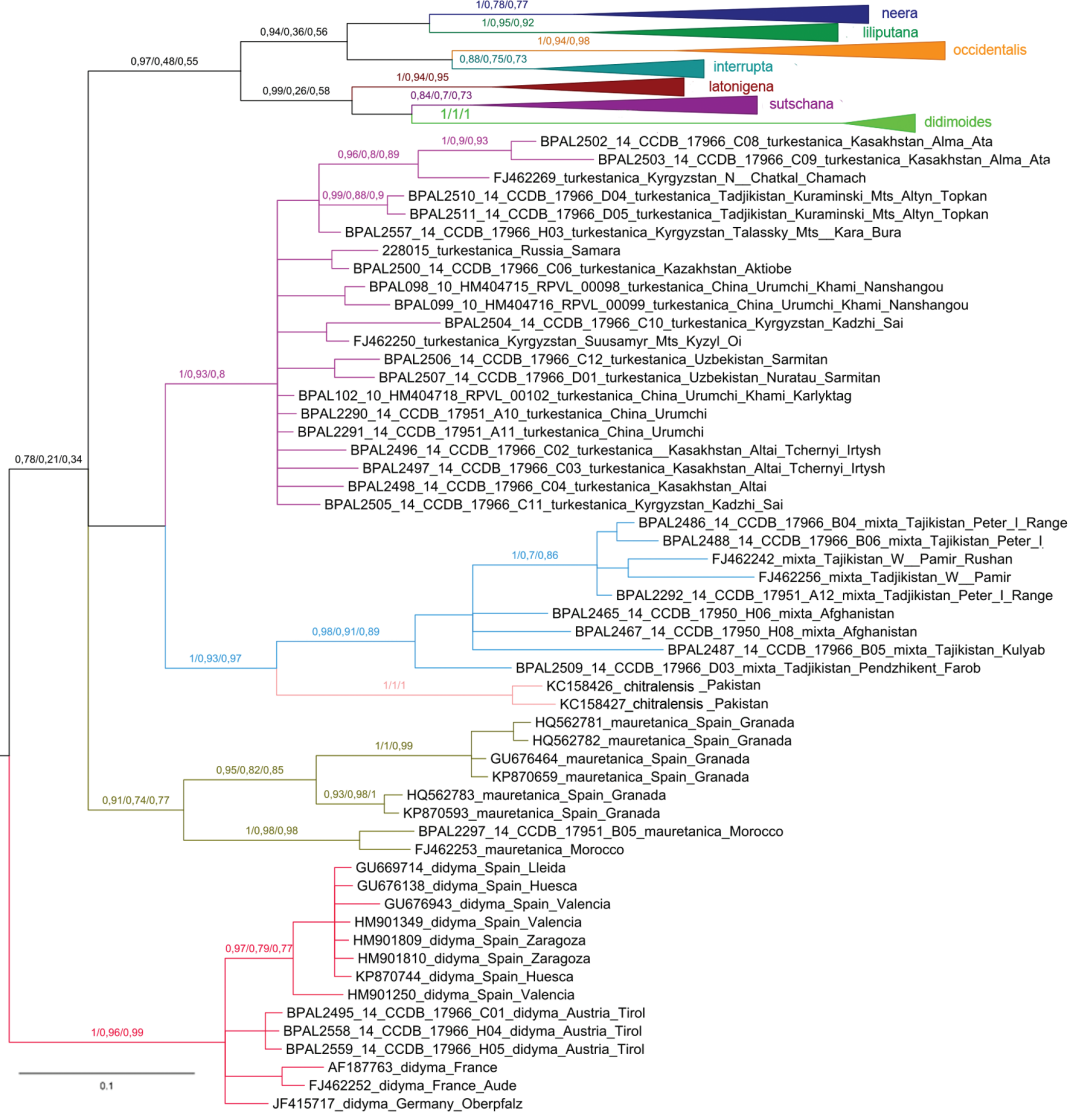
Fragment of the Bayesian tree of *Melitaea didyma* complex (haplogroups *turkestanica*, *mixta*, *chitralensis*, *mauretanica* and *didyma*) based on analysis of the *cytochrome oxidase subunit I*
(COI) gene. Numbers at nodes indicate Bayesian posterior probability/ML bootstrap/MP bootstrap values, with nonmatching clades using different analyses indicated by ‘-’. Scale bar = 0.1 substitutions per position.

**Figure 4.**
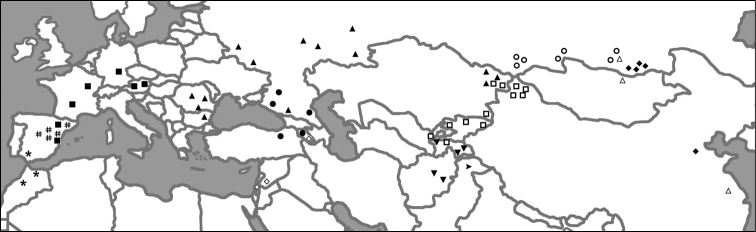
Distribution ranges of haplogroups *didyma* (■), *didymoides* (∆), *interrupta* (●), *latonigena* (○), *liliputana* (◊), *mauretanica* (*), *mixta* (▼), *neera* (▲), *occidentalis* (#), *sutschana* (♦), *turkestanica* (□) and *chitralensis* (►).

This analysis recovered the *Melitaea didyma* group as a strongly supported monophyletic clade (Fig. 1). Within this group many clades were well supported, whereas some of the relationships were not fully resolved (Figs 2 and 3). Within the complex we identified 12 differentiated major *COI* haplogroups. All of them showed a strict attachment to the localities (Fig. 4). Therefore in order to designate these haplogroups, we chose the oldest available name that was described from the area of each haplogroup: *Melitaea mauretanica* Oberthür, 1909, *Melitaea occidentalis* Staudinger, 1861, *Melitaea didyma* Esper, 1779, *Melitaea neera* Fischer de Waldheim, 1840, *Melitaea interrupta* Colenati, 1846, *Melitaea liliputana* Oberthür, 1909, *Melitaea turkestanica* Sheljuzhko, 1929, *Melitaea mixta* Evans, 1912, *Melitaea chitralensis* Moore, 1901, *Melitaea latonigena* Eversmann, 1847, *Melitaea didymoides* Eversmann, 1847 and *Melitaea sutschana* Staudinger, 1892 (Figs 2 and 3). The name *Melitaea liliputana* was selected for the Middle East populations of the *Melitaea didyma* complex. These populations have been known under the name *libanotica* Belter, 1934 in the literature (Larsen 1974, Benyamini 2002, Tshikolovets 2011). However, the name *liliputana* was preferred since ICZN states priority of the oldest available name (article 23, Principle of Priority).

The discovered haplogroups correspond to two traditionally recognized species (*Melitaea didymoides* and *Melitaea sutschana*) (Higgins 1941), to four taxa that were recognized as species relatively recently (*Melitaea latonigena*, *Melitaea interrupta*, *Melitaea chitralensis* and *Melitaea mixta*) (Lukhtanov and Kuznetsova 1989, Hesselbarth et al. 1995, Kolesnichenko 1999, Kolesnichenko et al. 2011), to five recognized subspecies (*Melitaea didyma occidentalis*, *Melitaea didyma didyma*, *Melitaea didyma neera*, *Melitaea didyma liliputana* and *Melitaea didyma turkestanica*) (Higgins 1941, Larsen 1974, Benyamini 2002, Tshikolovets 2011) and to one form (*Melitaea mauretanica*) whose status (subspecies or individual variations) is unclear (Higgins 1941).

There is good evidence based on analysis of morphology and observations of taxa in sympatry that *Melitaea didymoides*, *Melitaea sutschana*, *Melitaea latonigena*, *Melitaea interrupta*, *Melitaea chitralensis* and *Melitaea mixta* represent true biological species (Higgins 1941, Lukhtanov and Kuznetsova 1989, Hesselbarth et al. 1995, Kolesnichenko 1999, Kolesnichenko et al. 2011). Theoretically, the remainder of the *Melitaea didyma* complex can be interpreted as a single species *Melitaea didyma*. However, such an interpretation meets two difficulties. Firstly, such a lumping would result in a polyphyletic assemblage. Monophyly is the basic principle of phylogenetics and taxonomy. The majority of taxonomists currently believe that monophyly, in the narrow sense used by Hennig (Hennig 1966, Envall 2008, Hörandl and Stuessy 2010) is mandatory. Thus avoiding non-monophyletic groups and focusing on monophyletic entities is the preferable option in practical terms (Talavera et al. 2013b). The *COI* barcodes alone can provide weak evidence for monophyly of taxa since trees inferred from single markers sometimes display relationships that reflect the evolutionary histories of individual genes rather than the species being studied. Mitochondrial introgression (Zakharov et al. 2009) and *Wolbachia* infection (Ritter et al. 2013) can lead to additional bias in inferring phylogenetic relationships. Despite these limitations, we argue that, until not falsified, clusters based on DNA barcode monophyly represent preferable primary taxonomic hypotheses than the clusters based on para- or polyphyletic DNA barcode assemblages.

Secondly, the uncorrected p-distances between these taxa are high (from 1.3% between *neera* and *liliputana* to 3.9% between *liliputana* and *occidentalis*). Although some of them are lower than the ‘standard’ 2.7–3.0% DNA-barcoding threshold usually used for allopatric taxa as an indicator for their species distinctness (Lambert et al. 2005, Lukhtanov et al. 2015), even the lowest distances are comparable with those found between other six well recognized species. For example, distances between *interrrupta*, *latonigena* and *mixta* and their sympatric/parapatric non-conspecifics are 1.6-1.9% (Table 1).

**Table 1.** Minimal uncorrected *COI* p-distances between 12 major haplogroups of the *Melitaea didyma* species complex (%).

	1.	2.	3.	4.	5.	6.	7.	8.	9.	10.	11.
1. *neera*											
2. *liliputana*	1.3										
3. *occidentalis*	2.7	3.9									
4. *interrupta*	1.8	3	1.9								
5. *latonigena*	1.9	3.2	3.6	3.26							
6. *sutschana*	2.2	3.6	3	3.28	1.89						
7. *didymoides*	3.8	4.8	4.4	3	3.6	3.29					
8. *turkestanica*	1.6	2.7	2.4	2.43	2.16	2.73	3.89				
9. *mixta*	2.7	3.6	3	3.2	3.86	3.87	4.77	1.89			
10. *chitralensis*	4.3	4.7	4.6	4.1	4.3	4.3	5.2	3,2	2.4		
11. *mauretanica*	1.6	2.9	2.16	1.9	2.16	3	3.88	1.6	2.18	3.8	
12. *didyma*	1.9	3	2.73	2.4	2.44	3	4.48	1.6	3	3.3	1.61

Sympatry (or at least parapatry) (shown by green color) was demonstrated for the following taxa pairs: *mixta* and *turkestanica* (Kolesnichenko et al. 2011), *mixta* and *chitralensis* (Higgins 1941), *didymoides* and *sutschana* (Gorbunov 2001), *didymoides* and *latonigena* (Gorbunov 2001), *sutschana* and *latonigena* (Gorbunov 2001), *latonigena* and *neera* (Lukhtanov et al. 2007), *interrupta* and *neera* (parapatry in the North Caucasus, Tuzov and Churkin 2000) and *interrupta* and *liliputana* (parapatry in Armenia and Turkey, Hesselbarth et al. 1995). Here we also report an observation of parapatry between *neera* and *turkestanica* in South Altai and Zaisan valley in East Kazakhstan (shown by green color). In this area the distribution ranges of these taxa overlap, however, the taxa are separated ecologically: *Melitaea neera* is associated with the steppe biotopes and *Melitaea turkestanica* is associated with deserts. Sympatry was also found between haplogroups *occidentalis* and *didyma* sensu stricto in Spain (shown by yellow, Dincă et al. 2015). However, morphology and ecology of the bearers of these haplogroups were not analyzed in the contact zone. Therefore, evolutionary and taxonomic interpretation of this case of sympatry is difficult. It may represent sympatric distribution of two different species or may be a consequence of mitochondrial introgression between the allopatric pair *occidentalis*-*didyma*.

Finally, five of the six remaining haplogroups (*occidentalis*, *didyma* sensu stricto, *neera*, *liliputana* and *turkestanica*) are morphologically distinct and have been considered as separate taxonomic entities (subspecies) (Higgins 1941, Larsen 1974, Benyamini 2002, Tshikolovets 2011). Their monophyly with respect to the *COI* gene reinforces the conclusion that they represent independent lineages of evolution.

Therefore, we hypothesize that the *Melitaea didyma* complex is represented by the following 12 species that can be recognized by a phylogenetic species concept (Cracraft 1989, Coyne and Orr 2004) (taxa 1–5) and by both phylogenetic and biological species concepts (taxa 6–12):

1. *Melitaea liliputana* Oberthür, 1909 (Armenia, Turkey, Syria, Israel)
2. *Melitaea occidentalis* Staudinger, 1961 (Spain)
3. *Melitaea didyma* Esper, 1779 (west Europe)
4. *Melitaea neera* Fischer de Waldheim, 1840 (east Europe, north Caucasus, west Siberia, north Kazakhstan)
5. *Melitaea mauretanica* Oberthür, 1909 (north Africa, south Spain)
6. *Melitaea interrupta* Colenati, 1846 (Caucasus, Turkey, Iran)
7. *Melitaea turkestanica* Sheljuzhko, 1929 (Kazakhstan, Kyrgyzstan, Uzbekistan, Tajikistan, west China)
8. *Melitaea mixta* Evans, 1912 (Tajikistan, Pakistan, Afghanistan)
9. *Melitaea chitralensis* Moore, 1901 (north Pakistan)
10. *Melitaea latonigena* Eversmann, 1847 (Asian Russia, north-east Kazakhstan, Mongolia, north-west China)
11. *Melitaea didymoides* Eversmann, 1847 (Asian Russia, Mongolia, North China)
12. *Melitaea sutschana* Staudinger, 1892 (Far East Russia, Korea, North-East China)

## Supplementary materials

Supplementary material 1 Table S1 Data type: Excel table. Explanation note: Collection data of the samples sequenced in this study. This dataset is made available under the Open Database License (http://opendatacommons.org/licenses/odbl/1.0/). The Open Database License (ODbL) is a license agreement intended to allow users to freely share, modify, and use this Dataset while maintaining this same freedom for others, provided that the original source and author(s) are credited.Elena A. Pazhenkova, Evgeny V. Zakharov, Vladimir A. Lukhtanov

Supplementary material 2 Table S2 Data type: Excel table. Explanation note: List of the samples used in this study This dataset is made available under the Open Database License (http://opendatacommons.org/licenses/odbl/1.0/). The Open Database License (ODbL) is a license agreement intended to allow users to freely share, modify, and use this Dataset while maintaining this same freedom for others, provided that the original source and author(s) are credited.Elena A. Pazhenkova, Evgeny V. Zakharov, Vladimir A. Lukhtanov
